# Supplementary material for: Osmolality as a strong predictor of COVID-19 mortality and its possible links to other biomarkers
Source: PLoS One. 2025 Sep 16;20(9):e0331344. doi: 10.1371/journal.pone.0331344 (PMC12440178; doi:10.1371/journal.pone.0331344)
Supplement: Attachment — Submitted filename: response-june24-apr25.pdf [file pone.0331344.s001.pdf]

Responses to Editor's letter on April 22, 2024

Sirin Cetin, Ayse Ulgen, Hakan Sivgin, Meryem Cetin, Wentian Li

April 8, 2025

We do not think the Editor made a convincing case in the rejection letter on April 22, 2024 for our submission "Osmolality as a strong predictor of COVID-19 mortality or hospitalization and its possible links to other biomarkers". Both reviewers 1 and 3 expressed positive opinion on our submission (in their first round report on March 13, 2024): "good work" from reviewer-1; "the study presents some interesting findings" from reviewer-3. We wrote a point-by-point response to the three reviewers (sent to us on March 13, 2024), but we did not hear directly from the reviewers to our revisions on the letter on April 22, 2024, and we only got the following opinion from the Editor:

> "We appreciate your reply to the remarks made by the reviewers and editor. Nonetheless, the answer lacked significance in certain important areas. There was a breach of the PLoS One Journal protocol (abstract, introduction, etc.). Furthermore, there is a lack of proper referencing across the Methods, and Discussion sections (e.g., the definition of osmolality in the procedures, lines 81–86). These facts indicate that the manuscript is not suitable for publishing in PLoS One Journal."

These comments were mainly from the reviewer-2 in the first round report on March 13, 2024 which were repeated by the Editor, then repeated again in the second round (on April 22, 2024). We thought we responded these comments in our revision already. The Editor's letter made us question if our response letter was actually read by the Editor?

While we have been making the paper better since then (even beyond the reviewers' comments), we would like to appeal our case again by responding to Editor's April 22, 2024 comments here:

(On "There was a breach of the PLoS One Journal protocol (abstract, introduction, etc.)")

In our response letter in the second round, we wrote: "We use the PLoS LaTeX template so that these style changes have been made as a consequence: the tables are moved to close to

where these are cited; title is in lowercase (except the first letter); the abstract is made to the same level as other sections; line numbers are added to manuscript"

For protocol on abstract, we check all items in the requirement: (https://journals.plos.org/digitalhealth/s/submission-guidelines)

- not exceeding 300 words ✓
- should mention techniques used ✓
- do not apply background, methodology/finding, conclusion, in the heading, even though these concepts are used ✓
- do not include citation ✓
- do not include abbreviations ✓

For protocol on Introduction section, let us quote the PLoS instruction here: "The introduction should put the focus of the manuscript into a broader context. As you compose the Introduction, think of readers who are not experts in this field. Include a brief review of the key literature. If there are relevant controversies or disagreements in the field, they should be mentioned so that a non-expert reader can delve into these issues further. The Introduction should conclude with a brief statement of the overall aim of the experiments and a comment about whether that aim was achieved." Again, we are not clear why our Introduction violates the protocol.

Combining both the style (since we adopt the PLoS LaTeX template) and content (we do not see a violation of the instruction), the Editor did not provide enough detailed direction for us to move on.

(On "there is a lack of proper referencing across the Methods, and Discussion sections..)

In our response in the second round, we replied to reviewer-3: "We are perhaps the first is using either collider analysis or mediation analysis in the osmolality context, so there is no references that we are aware of that are directly relevant. The logic of using the mediation analysis is mentioned at the beginning of this Response (to both editor and reviewers). We have added one textbook reference on causal inference where the collider analysis is mentioned (Hernan and Robins,2020). For osmolality as a predictor for COVID-19 severity, we had one reference already (Ramesh et al, 2021). In fact, a motivation for writing this paper is a lack of osmolality-severity work in the literature, and we want to fill this gap".

Then, in our response to reviewer-2, we wrote "As mentioned in our response to Reviewer-3 [there was a typo before, we wrote reviewer-2 instead], we are perhaps the first to use collider

analysis and mediation analysis in this context. Consequently, it is relatively hard to find relevant papers in the literature."

These explained a lack of reference in our submission. In the current version, since we realized that a lack of reference may make it harder to understand our method, we have added as much more information as possible in the paper itself, for people who had no prior exposure to causal analysis. We have completely rewritten any part (in Introduction, Method, Result, Discussion) concerning the construction of a proposed causal model involving osmolality (i.e. Fig.3). In particular, we have added a new subsection and a new figure (Fig.2) to explain, step-by-step, our pipeline. In this way, readers can use our pipeline to follow what we did, instead of searching the causal analysis literature.

(On "..(e.g., the definition of osmolality in the procedures, lines 81–86)")

This sentence from the Editor indicates that our response letter was not read by anyone from the journal.

In our response letter in the revision stage, we wrote in response to reviewer-2: "We have a subsection in 'Data and Method': "definition of osmolality", where the formula used is defined. We need further clarification from the reviewer to know what the question is."

We have cited a reference for this definition of osmolality in the Introduction section (second paragraph, ref.[24]:

> M Rasouli (2016), Basic concepts and practical equations on osmolality: biochemical approach, Clin. Biochem., 49:936-941.

This reference was cited in the Introduction section in the first round, whereas cited in both Introduction and Method section in the second round, to make it easy to find.

Besides these responses to the Editor, we have also made other changes to improve the paper, such as: *more focus on mortality instead of (surviving) hospitalization by removing the work "hospitalization" from the title, as the association signal with mortality is stronger; *using the same row order for Tables 1 and 2 to make a comparison between the two easier; *emphasize a take-home lesson of the paper, that the use of osmolality in prognostics may do better than using its components; *clarify that odds-ratio(OR) value can change by a variable transformation (e.g. log transformation).
